# The wide world of biology in 2019

**DOI:** 10.1038/s42003-020-0783-x

**Published:** 2020-02-10

## Abstract

As we celebrate our second year of publishing, we look back on some highlights from 2019 in *Communications Biology* and consider what it means to study biology.

This week we published our 2nd anniversary collection, including editors’ picks of some of our best research from 2019. Limiting the number of articles chosen by each editor to fewer than six was no easy feat. We published 435 primary research Articles in 2019—more than twice as many as the previous year—and we are proud of each one. We are also extremely grateful to the 2530 reviewers who critiqued and improved all of the papers we sent to review. See the accompanying editorial^1^ for more about our reviewers in 2019.

Each editor who contributed to the anniversary collection used their own criteria. For example, the papers may have been ones that they found particularly insightful, fascinating, or elegant. Or perhaps they felt the topic was of particular importance. Whatever the motivation, the 40-plus articles chosen for our anniversary collection highlight the breadth and diversity of the biological sciences.

Our most cited and most downloaded articles from 2019 give a glimpse of this diversity. Among our most cited papers are novel tools for single-particle electron cryomicroscopy and imaging intracellular K^+^ dynamics. There are also breakthroughs in banana breeding and RNA editing. Among our most downloaded articles is a report of a tiny tyrannosauroid and a study warning of the dangers of plastic waste to oxygen-producing microbes in our oceans.

Some of our most-read articles from the past year are reviews and commentaries. For example, this scientometric review of genome-wide association studies has already been cited over 25 times and downloaded more than 19,000 times. Two ‘After the Paper’ Comment articles published as part of our first year anniversary collection—on wheat stem rust and sea turtle fibropapillomas—have each been downloaded around 40,000 times. And a recent Comment article on partnerships between scientists and graphic artists received wide attention online and has also been downloaded more than 19,000 times.

Biologists are not just one thing. In fact, few biologists consider themselves simply *biologists*. They are molecular biologists, microbiologists, ecologists, evolutionary biologists, geneticists, cell biologists, plant biologists, and—well, you get the picture. But they all have the same goal: to understand life. This is why we feel so strongly about the need for journals like *Communications Biology*. The journal is a place where biologists don’t need to define themselves by any one sub-area of biology. They can simply be biologists.

The journal is a place where biologists don’t need to define themselves by any one sub-area of biology. They can simply be biologists.

In recent years, some of the hottest areas of biology were shared between subdisciplines. Single-cell omics has led to groundbreaking findings in cancer, immunology, genetics, metabolism, and neuroscience. Understanding the microbiome continues to be a focus of fields as seemingly different as microbiology, neurobiology, and plant sciences. Advances in imaging have contributed to nearly all areas of the life sciences. In fact, when you move past the subject area-specific jargon, it seems that the many different flavors of biology are not so isolated from each other after all.

It’s true that few scientists now read any journal from cover to cover (we don’t even have covers) and most literature is discovered by focused searches on a specific topic. Even so, we encourage you to take a few minutes each week or month to find something totally unexpected that can spark an idea offer a clue that will help in your own research. And we hope that you will consider scrolling through our latest articles and collections when you do so.
